# Developing a Method to Test the Validity of 24 Hour Time Use Diaries Using Wearable Cameras: A Feasibility Pilot

**DOI:** 10.1371/journal.pone.0142198

**Published:** 2015-12-03

**Authors:** Paul Kelly, Emma Thomas, Aiden Doherty, Teresa Harms, Órlaith Burke, Jonathan Gershuny, Charlie Foster

**Affiliations:** 1 Physical Activity for Health Research Centre, Institute for Sport, Physical Education and Health Sciences, University of Edinburgh, Edinburgh, United Kingdom; 2 British Heart Foundation Centre on Population Approaches for Non-Communicable Disease Prevention, Nuffield Department of Population Health, University of Oxford, Oxford, Oxfordshire, United Kingdom; 3 Centre for Time Use Research, Department of Sociology, University of Oxford, Oxford, Oxfordshire, United Kingdom; 4 Nuffield Department of Population Health, University of Oxford, Oxford, Oxfordshire, United Kingdom; Indiana University, UNITED STATES

## Abstract

Self-report time use diaries collect a continuous sequenced record of daily activities but the validity of the data they produce is uncertain. This study tests the feasibility of using wearable cameras to generate, through image prompted interview, reconstructed 'near-objective' data to assess their validity. 16 volunteers completed the Harmonised European Time Use Survey (HETUS) diary and used an Autographer wearable camera (recording images at approximately 15 second intervals) for the waking hours of the same 24-hour period. Participants then completed an interview in which visual images were used as prompts to reconstruct a record of activities for comparison with the diary record. 14 participants complied with the full collection protocol. We compared time use and number of discrete activities from the diary and camera records (using 10 classifications of activity). In terms of aggregate totals of daily time use we found no significant difference between the diary and camera data. In terms of number of discrete activities, participants reported a mean of 19.2 activities per day in the diaries, while image prompted interviews revealed 41.1 activities per day. The visualisations of the individual activity sequences reveal some potentially important differences between the two record types, which will be explored at the next project stage. This study demonstrates the feasibility of using wearable cameras to reconstruct time use through image prompted interview in order to test the concurrent validity of 24-hour activity time-use budgets. In future we need a suitably powered study to assess the validity and reliability of 24-hour time use diaries.

## Introduction

### Time Use Diaries

We are not in general aware of the quantities of time we devote to the activities of our normal days. Time use diaries are therefore used by sociologists and economists to document the hours and minutes spent on various daily activities such as paid work, watching television, exercising, other leisure activities, sleeping, child and elder care and household work [1,2]. They are now increasingly used by health researchers, insofar as these same activities are also differentially associated with levels of exercise or sedentary behaviour, as well as nutritional behaviour (meals versus ‘browsing’), relaxation and sleep patterns [3–5].

Random samples of time use diaries allow cross-sectional and inter-temporal comparisons of the allocation of time across different populations. Time use diary data also provide information on how a given activity is placed sequentially in relation to other leisure and non-leisure activities, as well as the geographical location of activities, the time at which they occurred, and the sequence of temporal-spatial locations [6]. These data allow researchers to explore important trends, correlates and determinants in behaviour. This in turn helps us to better understand the implications of social change and identify areas for policy and intervention, for example around gender equality or health outcomes.

These data are collected using self-reported ‘tomorrow’ or ‘yesterday’ instruments. Interviewers leave ‘tomorrow’ diaries behind for the respondent to complete on the following day or set of days. ‘Yesterday’ diaries require respondent to recall and reconstruct recent events as the interviewer asks about the activities in which she or he engaged on the previous day. The Harmonized European Time Use Survey (HETUS) design (a tomorrow diary) has been used in over 40 national surveys over the last 15 years and provides a ‘gold standard’ for both cross-national and long-run historical comparisons [7].

Self-report measures are frequently employed as they have many advantages over objective measures including relative cost, scalability, and the ability to capture contextual data. In contrast, the validity and reliability of self-report measures, including diaries, have been debated in relation to problems of both recall and social desirability effects [8]. Fixed cameras were first used in the 1970s to provide ‘objective’ measures of specific types of activities [9]. Research comparing self-report diaries with other measures, including Experience Sampling Method and stylised reports, generally supports the use of diaries to estimate aggregate time allocation [10]. However, comparison to more objective measures will allow researchers to take more confidence in their time-use diary data, and also to understand any systematic biases that may be present.

### Wearable Cameras

Wearable cameras provide a cheaper proxy for the criterion method of continuous direct researcher observation of behaviour or activity, covering a much fuller range of activities than did fixed camera studies [11–15]. They have previously been used to record, classify and quantify travel [5,13,16], sedentary behaviour [14], physical activity [11] and diet [12,17,18]. They record up to 4000 ‘wearer’s perspective’ images per day (at approximately 15 second intervals) which can be aggregated into counts of activity durations and sequences [15]. The image data can also be used as recall prompts for participant ‘reconstruction’ interviews [12,18] allowing the participant to verify activity types, purposes and locations. In the context of travel, they have been shown to have near perfect agreement with direct researcher observation [19].

Wearable cameras have the advantage of passively recording more information, with greater accuracy than self-reported diaries. In contrast self-report diaries have the advantage of scale, cost, ease of coding and analysis, fewer privacy concerns and greater acceptability. Therefore it is appropriate to use wearable cameras on a small scale to better understand the data produced by self-report diaries. This will provide understanding of the nature of data collected in population based studies using self-report diaries.

The aim of this study was to test the feasibility of using wearable camera images to reconstruct an ‘objective’ record of daily time use that can be used to assess the concurrent validity of self-reported diary records. We had the following research questions:

1. Would participants comply with a measurement protocol that involves wearing a wearable camera for 10–14 hours (the waking hours of the measurement day) while following the HETUS 24 hour self-reported time use diary protocol?
2. Would we be able to use the image data as a prompt for participants to reconstruct the activities of the measurement day in a researcher-guided interview?
3. What did the camera data tell us about the validity of the concurrent self-reported time use data?

## Methods

### Ethics Approval

This study received ethical approval from University of Oxford (Inter-Divisional Research Ethics Committee (IDREC) reference number: SSD/CUREC1A/13-262). Participants provided prior written consent and all signed consent forms were retained. This process was approved by IDREC. We used specific ethical guidance for wearable camera research to inform the ethical development of protocols. [20].

Wearable cameras present additional privacy considerations beyond standard observational diary research. The privacy of participants or third parties can be threatened if images are made publically available through lost devices or miss-use of social media. Therefore all devices were configured such that only the research team could access images, and images from lost devices could not be accessed by anyone else. Further, all research staff are given additional ethical training in handling, storage and transfer of images. Participants also have the option to view and delete any or all of their images before the research team view them in case of sensitive, embarrassing or private images.

### Recruitment

All participants were volunteers from Oxford, Oxfordshire, UK and recruited from existing academic networks (e.g. participants from previous research studies, departmental colleagues not working on this project, etc) by email or in person.

Each participant was provided with an information booklet which discussed the study in detail. After 24 hours, the participant was followed-up (either by email or face-to-face) to see if they wished to take part and if so, a ‘set-up’ meeting was arranged. During the set-up meeting, the study was discussed in detail, instructions provided on how to wear the devices, and if still wishing to partake, the consent form was completed. A study day was decided and the researchers sent a text in the morning as a reminder to put the camera on. Participants were encouraged to contact the researchers if they had any questions or concerns during the study day.

A sample of 16 participants of various ages was considered appropriate, given the exploratory nature of this pilot study. Participants were given an Information booklet outlining the purpose of the study and their involvement in it.

### Study Protocol and Study Visit

Having volunteered to take part, members of the research team guided participants through the Participant Information booklet, giving them an opportunity to ask questions before providing their informed consent. On the day before the measurement day, participants were given a diary, wearable camera and accelerometer and completed a short demographic questionnaire. This process took no longer than 20 minutes.

### Time Use Diary

The UK National Time Use Survey 2014–15, currently in the field, deploys the HETUS time use diary. This is completed by respondents in their own words, in 10-minute intervals for 24 hours (04.00am– 04.00am). Diarists record their primary (main) activity, any secondary (simultaneous) activity, location or mode of transport, who they were with, whether a smart phone, computer or tablet were used in the activity, and how much they enjoyed the activity on a scale of 1–7. The same instrument was used in this study though we did not analyse activity enjoyment for this study.

### Wearable Camera Protocol

**Fig 1:**
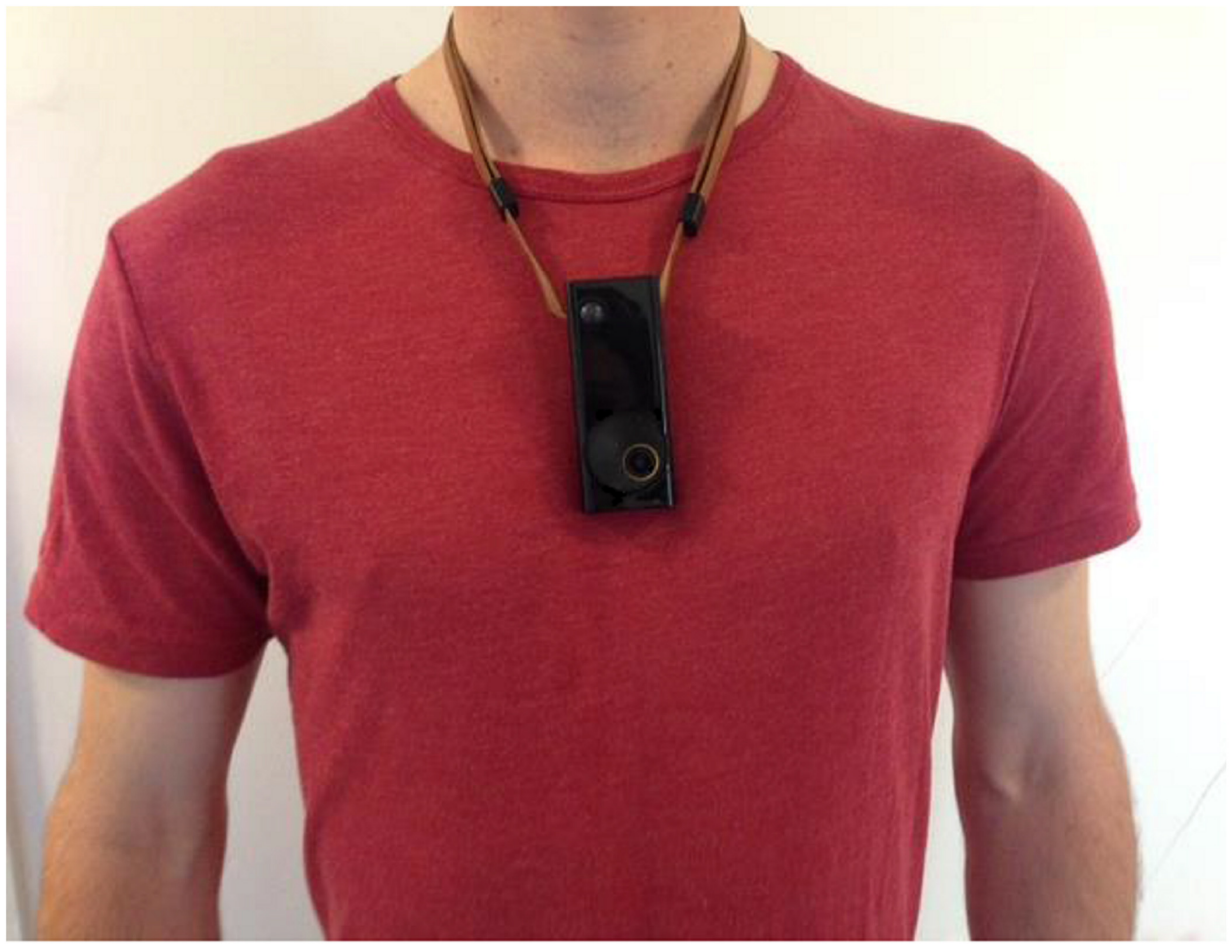
The Autographer wearable camera on adjustable lanyard.

Participants wore an Autographer camera on a lanyard during waking hours of the collection day (Fig 1). They were assured that they could remove the device or stop recording at any time if they were feeling uncomfortable or in a location where wearing the camera was inappropriate (e.g. a changing room).

To minimise reactivity in reporting (i.e. more precise diary reporting than usual diary samples as a result of knowing accuracy was the subject of the study), participants were told that the main purpose of the study was a general comparison of diary and camera images, rather than assessing the accuracy of the diary data. Participants were given the option of receiving a reminder email or SMS text message to prompt them to turn on and wear the camera on the collection day.

The cameras were configured so that only the researchers could download the images and participants or other third parties could not access or save their own copies. All protocols were in accordance with an ethical framework developed specifically for wearable camera research [20].

### Accelerometer Protocol

Participants were also asked to wear a wrist worn ‘activity watch’ (GENEActiv accelerometer) [21] from the end of the study visit until the morning after the data collection day. These data were not used in the current analysis.

### Return of Devices and Data and Satisfaction Survey

Members of the research team collected the study instruments and conducted a ‘reconstruction’ interview no more than four days after the data collection day. After downloading the Autographer images, researchers invited participants to delete any images they wished (in private, if preferred). While the images were downloading (which takes about 5 minutes) researchers collected a short questionnaire, extended from the work of Caprani and colleagues [22], to assess participants’ experience of the data collection process. Additional questions about the experience of the reconstruction interview were completed subsequently. The diaries were also collected and checked for completion.

### Reconstruction Interview

Once participants had deleted any images, a member of the research team guided them through the camera images using the Doherty Browser [23], asking them to describe their activities recorded in successive images. Participants were asked to nominate the primary (main) activity when two or more activities were occurring simultaneously (e.g. eating and watching television).

Participants were also asked what time they woke up and went to sleep, what activities they engaged in before and after activating the camera, and (if they were willing to reveal this) what they were doing during any period when the camera was off, covered or obscured. The researcher noted activities that were reported during these periods as ‘non-wear-time’ or ‘obscured’; if the participant was unwilling to respond, these activities were noted as ‘activity un-reported’. The interviews were audio recorded for later reference when coding the images for the reconstruction day.

### Image Annotation and Criterion Day Construction

Using the reconstruction interview notes and the Autographer images, one researcher annotated the activities in the image browser software, using the same HETUS coding framework. This provided a near-objective ‘yesterday’ record of activities conducted during waking hours while the camera was in use. Sleep and waking times and activities before and after camera activation were manually added to the yesterday data file from the reconstruction interview notes to create a full 24-hour record.

### Diary Coding

Both the ‘tomorrow’ respondent diary and the ‘yesterday’ reconstruction data were coded using the HETUS coding frame (UK version). A time use episode in the HETUS database is defined by four substantive domains: primary and secondary activities coded to a 3-digit classification; location/means of transport coded to 11 categories; ‘with whom’ (coded to 8 categories); and a temporal identifier. The temporal identifier holds information on the time when episodes start and end [7].

The coding process results in two similarly structured time use data files for each participant, corresponding respectively to the ‘yesterday’ image-based reconstruction and the ‘tomorrow’ self-report diary data. Each of the files has a list of activities with associated HETUS activity code, location and start- and end-times.

### Data Analysis

Any sample of daily time diaries allows three types of *aggregate* time use statistics to be calculated: (1) means of time spent in each category of main and secondary activity at the group or sample level (Sample mean time, T_S_); (2) ‘participation rates’ or proportions of respondents who engage in each activity; and (3) ‘participant’s means’ of time calculated for those respondent diarists who actually engage in the activity during the diary day (Participants’ mean time, t_p_). The first of these statistics are the product of the second and the third. We use all three types of statistic in our discussion of the relationship between the aggregate time estimation from camera and self-report diary records to allow assessment of the group and also the subset within the group that engage in a given activity.

For example, if we take an activity such as driving and assume a ‘participation rate’ of 50% (i.e. half of the sample engage in this activity) for 1 hour per day each; the ‘participants’ mean time’ will be 1 hour, while the ‘sample mean time’ will be 0.5 hours. This is a useful comparison for both common and uncommon activities.

We compared these statistics calculated from the days as reconstructed from the camera records, and from the time-use diary. We assessed the agreement between diary and reconstructed day records using *Levene’s test for equality of variance* (estimated in SPSS).

Time diaries also allow the calculation of sequence vectors which show the succession of activities through the day, as well as sequence statistics which summarize the similarity of pairs of sequences. We generated concurrent sequence plots for each participant that were analysed descriptively.

## Results

### Research Question 1—Compliance with the measurement protocol

#### Data collection and coding

Data collection and reconstruction interviews took place in March-July 2014. Return study visits comprising diary and device data return and the reconstruction interview lasted between 40 and 70 minutes per participant. Image annotation using the interview data, and generation of the reconstructed days took place in August-September 2014. Each reconstruction day took approximately three hours to build. Self-report time use diaries were also coded in August-September using standard HETUS protocols [7].

#### Participants and compliance

Overall, 16 participants were invited to take part in the pilot. Compliance with the protocol was 87.5%; 14 from 16 participants returning image and diary data and completing the reconstruction interview within 4 days of collection. Table 1 shows the participant demographics for the 14 participants who complied with the protocol. The educational status of the sample was high, reflecting high university-based recruitment. The age range of the participants was 21–58 years, with a higher proportion of females (77%).

**Table 1:** Participant demographics and image collection results for n = 14 who completed the study protocol.

Participants n (%)	14	(87.5%)
Male	4	(28.6%)
Female	10	(71.4%)
Educational status		
- completed undergraduate degree	4	(28.6%)
- completed graduate degree	10	(71.4%)
Age (mean in years)	33.5	
Range (years)	21–58	
Standard Deviation	10.28	
Mean number of images collected per participant (sd) over a 24 hr period	2462 (295)	
Mean duration of image data over a 24 hour period in minutes (sd)	985.6 (192.2) minutes	
Mean duration of image data over a 24 hour period in hours and minutes (sd)	16h 42min (3h 20min)	

The two participants that failed to complete the protocol were male aged 31 and 65 years. Both completed the day of data collection and returned all the devices, but were unavailable to complete the reconstruction interview due to their time commitments.

As part of the compliance assessment we asked participants to complete satisfaction surveys of their experience. Participants reported satisfaction with the level of information provided on the study and devices and also reported that they did not believe they had changed their behaviour as a result of wearing the devices. Participants were not concerned by the amount of time they were required to wear the camera and accelerometer.

The most frequently reported issue of wearing the camera was excessive movement (swinging on lanyard) during physical activity. Some participants resolved this by using the camera clip while others chose to remove the device during active movement. Privacy was an additional reason for the periods of removal of the device (e.g., reading emails, breastfeeding a child). Participants reported that the length of time for the return visit (40–70 minutes) was acceptable.

Key findings to research question 1 are summarised in Box 1.

**Box 1**

**Key findings in relation to research question 1** The data from the pilot study suggest that participants found the study protocol acceptable and were generally happy to comply with the camera and diary instructions. They were able to wear the camera during their daily routine, with minimal instances of removal or camera deactivation. They were also able to concurrently complete a standard self-report time use diary.

### Research Question 2 –Using image data to guide the reconstruction interviews

#### Reconstruction Interviews

**Fig 2:**
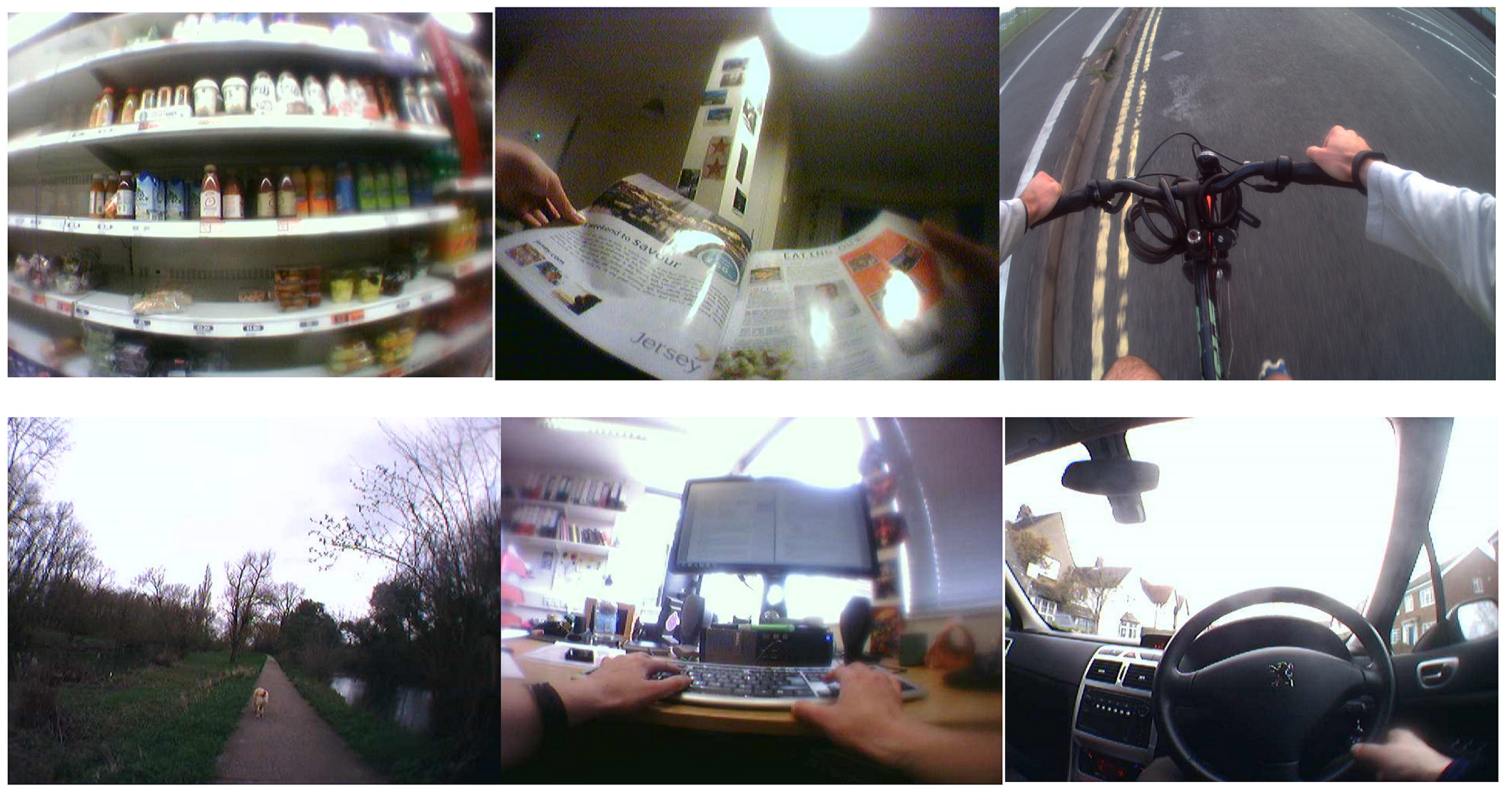
Sample of participants Autographer images from Pilot Study. Clockwise (from top left) these show the following activities with their appropriate HETUS Level one codes: food shopping (3: Household and family care), reading a magazine (8: Mass media), cycling (9: Travel), driving (9:Travel), computer use (1: Employment), and walking (6: Sports and outdoor activities).

A sample of collected images and their associated HETUS level one codes are shown in Fig 2. During the reconstruction interview, participants looked through the images and identified their daily activities and times of transition from one activity to the next. The summarized data from the images are shown in Table 1. Respondents were also able to report waking and going to bed times (the accelerometry data will be used to provide an objective time referent for these in the next stage of the project) and were asked to recall activities conducted during non-wear-time, using available images from before or after as prompts.

The mean duration of waking image data was 16h 42 min (equivalent to waking at 6.00am and retiring to bed at 10.40pm), which represented longer waking days than we expected. Participants reported reasons for non-wear for the following activities: personal care (e.g. washing, dressing and toileting); child care (e.g. attending school events); working on confidential information (e.g. writing emails); resting and time out (e.g. just watching TV); and because others were not comfortable with the camera (e.g. partners or spouse or work colleagues).

Key findings to research question 2 are summarised in Box 2.

**Box 2**

**Key findings in relation to research question 2** Participants (n = 14) returned a mean of 2462 (sd = 295) images per day, representing a mean waking day of 16h 42min. Participants were able to use the images as prompts to objectively report their primary activity during waking hours, and transitions between activities. Using data from the reconstruction interviews we were able to successfully reconstruct the daily activities over a 24 hour period for 14 of the 16 participants.

### Research Question 3—Using cameras to validate self-report time use data

#### Comparing a reconstructed criterion day to the concurrent self-reported day

The image data (combined with interview-reported waking and going to bed times and activities during non-wear time) produce near-objective records of 24-hour time use which we describe as ‘reconstructed days’. These revealed a mean of 41.1 independent ‘Level 1’ HETUS activities per day (standard deviation 17.2), with mean durations ranging from 0.36 hours to 10.08 hours. The self-report time use diaries for the same 14 participants revealed a mean of 19.2 independent ‘Level 1’ HETUS activities per day (standard deviation 6.8), with mean activity-type durations ranging from 0.36 hours to 10.14 hours. Therefore the image-reconstructed day captured more than one and a half times as many activities compared with those reported in the self-report diary, with generally lower mean durations of activity bouts.

Table 2 shows the sample means of time (T_s_; minutes per day) in the 10 single-digit HETUS activities, as well as the N of participants in each activity and the participant mean times (t_p_) in these activities. The sample means of time in activities in general look remarkably similar in the two sorts of record (a result consistent with that from a previous larger scale application of these methods focusing on travel alone [5,13,16]). Under these circumstances, and given the small number of observations in the pilot sample, it seems more appropriate to focus on a comparison of the variability of the two samples. The Levene Test for equality of variances provides no grounds, on the basis of this pilot sample at least, for suspecting a significant difference in the variability in the samples.

**Table 2:** Comparison of minutes assigned to HETUS 1 digit activities by camera and diary day (Levene's Test for Equality of Variances: no significant differences).

	Sample mean time (T_S_)				Participants' mean time (t_p_)			
	Mean minutes	sd	prob. same	N	Meanmins	sd	prob. same	N
Personal Care (including sleep)								
camera	604.3	108.0	-	14	604.3	108.2	-	14
diary	608.5	102.0	0.867	14	608.5	101.7	0.867	14
Employment								
camera	333.1	224.0	-	14	424.0	151.8	-	11
diary	350.7	233.0	0.770	14	409.2	195.1	0.522	12
Study								
camera	21.3	53.4	-	14	99.3	83.2	-	3
diary	21.4	51.1	0.988	14	100.0	72.1	0.742	3
Household and family care								
camera	132.6	90.0	-	14	132.6	90.0	-	14
diary	133.6	112.0	0.455	14	133.6	112.3	0.455	14
Voluntary work and meetings								
camera	0.0	0	-	14	-	-	-	-
diary	0.0	0	-	14	-	-	-	-
Social life and entertainment								
camera	87.0	112.0	-	14	93.7	113.8	-	13
diary	86.4	135.0	0.537	14	121.0	146.8	0.448	10
Sports and outdoor activities								
camera	29.4	39.0	-	14	58.9	35.8	-	7
diary	23.6	32.7	0.442	14	55.0	26.6	0.23	6
Hobbies and computing								
camera	52.1	61.2	-	14	66.3	61.9	-	11
diary	26.4	34.3	0.205	14	52.9	30.4	0.187	7
Mass media								
camera	84.5	81.3	-	14	91.0	80.8	-	13
diary	100.7	113.0	0.173	14	141.0	110.1	0.132	10
Travel and unspecified time use								
camera	95.6	45.5	-	14	95.6	45.5	-	14
diary	88.6	58.7	0.356	14	95.4	55.0	0.426	13
*Combined screen time (Hobbies and computing + Mass media)*								
*camera*	*136*.*6*	*110*	-	*14*	*147*.*1*	*106*.*5*	-	*13*
*diary*	*127*.*1*	*117*	*0*.*600*	*14*	*148*.*3*	*113*.*0*	*0*.*711*	*12*

However, there are big differences between the camera and diary means of time in two categories: ‘hobbies/computing’ and ‘media’. The camera records show much more time (52 versus 26 minutes) in ‘hobbies and computing’ (which almost totally comprises computer use), whereas the self-report diaries contain much more television watching (141 minutes compared with 91 minutes for the camera-based mean). While 11 respondents participated in hobbies/computing according to the camera images, only 7 reported this in their diaries.

Most of the difference between the two separate categories disappears once we combine the computing and television-viewing categories into ‘Combined screen time’ (in *italics* at the bottom of the table). The participants’ time estimates differ by just one minute. We believe that respondents watching television programmes on their computers recorded television in their diaries, whereas the coders using the camera records saw simply the computer use.

**Fig 3:**
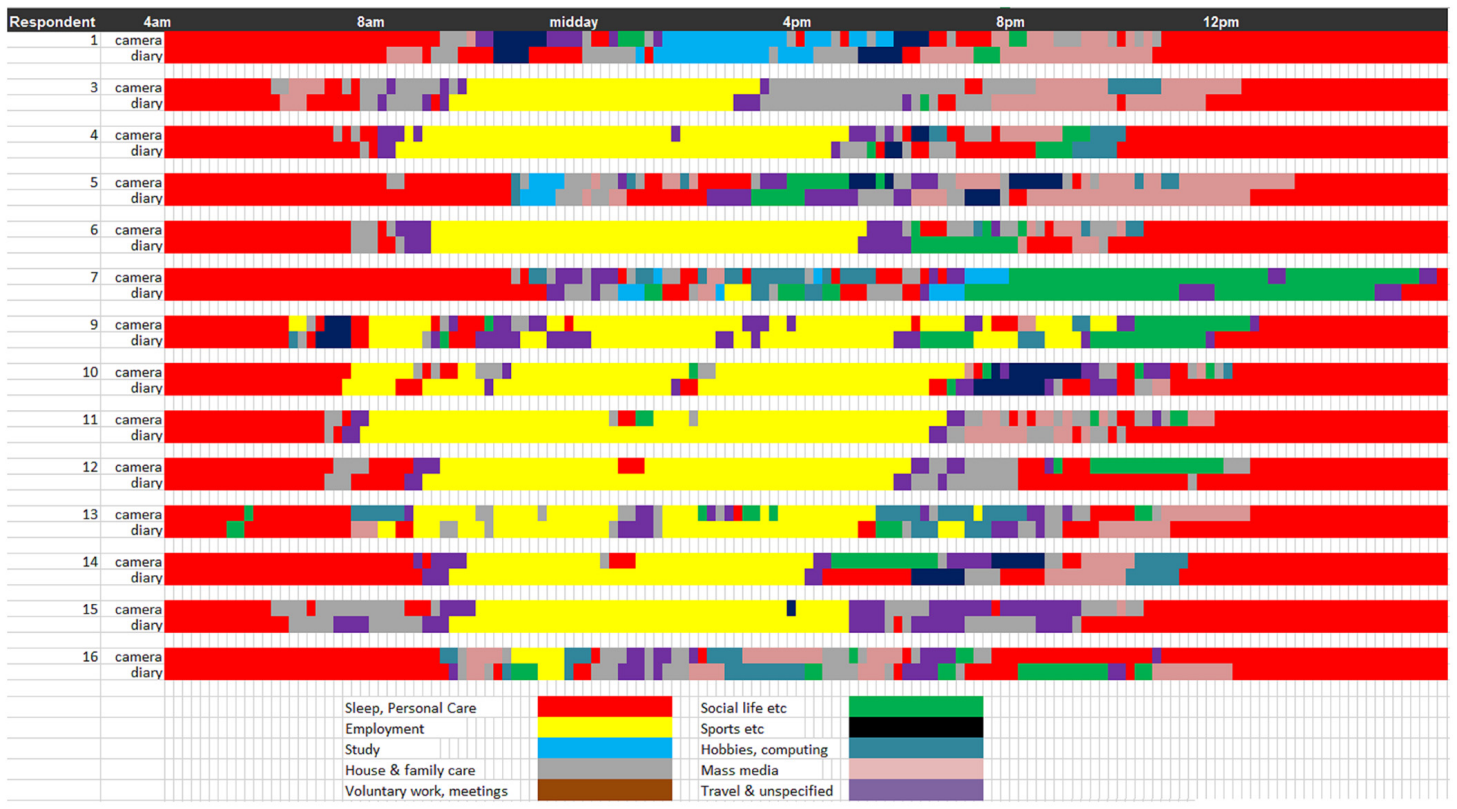
Sequence plot comparison for each participant (n = 14) of camera and diary measures (single digit activity classification).

Fig 3 provides a somewhat different perspective, displaying the activity sequences for each individual participant and comparing the reports in the reconstruction interview (camera record) and the diaries. Superficially, the pairs look rather different. Estimating the total durations visually, we immediately see that the time devoted to particular activities differs substantially between the camera and diary accounts, as well as some temporal mismatches in activity type for some respondents.

The overall similarity in mean times displayed in Table 2 suggests that contrasts between the two accounts of the same day for a particular person get averaged-out when we compare the diary accounts *overall* with the reconstruction interview accounts *overall*.

For example, respondent 14 reports more sleep in the time-use diary than the reconstruction interview, whereas for respondent 16, the reverse is true. Such differences are averaged-out in the aggregate totals in the type of analysis displayed in Table 2. An additional complexity emerges when we take note of the multiple activities, recorded most frequently in the self-report diaries. It seems that respondent 16 spent much of the time in bed, but reading rather than sleeping or resting. This potentially explains the extra ‘sleep’ time in in the camera-based account; much of the relevant period was recorded as reading in the time use diary. The diary straightforwardly clarifies the reason for the discrepancy: reading is reported as the primary activity by the diarist, resting is reported as the secondary.

Key findings to research question 3 are summarised in Box 3.

**Box 3**

**Key findings in relation to research question 3** The reconstructed pilot data suggest that participants report fewer HETUS Level 1 events (discrete periods devoted to particular activities) than were detected during the interview reconstruction process. The mean duration of each of the discrete diary events was proportionately higher than that found in the camera-based records. Nevertheless, the aggregate totals of elapsed time (durations) devoted to each of the activities across the camera and diary subsamples are strikingly similar. The sequence plot shows that while overall mean activity durations agree, there can be large disagreement in when activities happened, the order they occurred and overlapping or interrupting activities.

## Discussion

Self-completed *tomorrow* and *yesterday* diaries are the most commonly used field instrument in time-use research. This study has demonstrated the feasibility and acceptability of a wearable camera method for assessing the validity of the data these diaries produce. While not a replacement for 24 hour time-use diaries, it is a validation method for small scale samples that is likely to be less resource intensive and therefore more scalable than direct researcher observation, without sacrificing substantial objectivity.

Our findings suggest that 24 hour self-report time use data may under-estimate the number of discrete events on any given day. However, based on the preliminary analysis using the small pilot sample, self-report diaries provide valid mean durations and proportional distributions of time budgets across the day.

### Comparison to Literature

While wearable camera-based studies have been conducted to validate self-reported travel [5,16] and food intake [12,18], this is the first study to report a method for assessing 24-hour time use and activity budgets. It is also the first non-memory based study to use images as a prompt for participant recall of activities, which is likely to be superior to researchers viewing only the images without the participant present.

### Possible Explanations and Mechanisms

Image-led reconstruction intervals enable participants to report their primary activity with a time-stamped visual aid. Conversely, the HETUS diary is restricted to 10 minute intervals, so the coarser blocks of reporting time within the diary is likely to result in fewer reported activities overall. The images act as an ‘aide memiore’ known to assist in behavioural recall [24] whereas the diary relies on the participants to actively self-report their activities throughout the day. Reliance on diary completion can be problematic. The diarists may not be able to complete the diary regularly throughout the day for various reasons (e.g. work or child care commitments, inability to carry the diary around). Retrospective recording of events is likely to result in memory recall and perception difficulties. The automated nature of image collection removes reliance on the participants’ recall abilities.

### Implications

These results are not sufficient to provide implications for the use of self-reported time use data. However, they demonstrate that the method is feasible and that a full scale study is possible. Similar results emerging from a medium scale (n = 150 respondent, generalizable population) study will provide valuable evidence for further validating the diary method. It may also be possible to investigate reliability measurement properties. Sequence plots, and other sequences methods, may illuminate the mechanisms that explain apparent differences between the more objective camera method, and the more subjective diary accounts.

24 hour time use diaries could be a valuable source of historical trend data on daily energy expenditure (through assignment of metabolic equivalents of tasks (METS)) and reflecting changes in occupation and leisure time physical activity. Our study and method offers a means to examine such data with confidence in any derived MET based estimates.

Finally our method offers researchers in the health sciences behavioural specificity and context in conjunction with devices such as accelerometers to estimate and measure physical activity behaviours. The combination of wearable camera with interview methods will provide a solid empirical foundation that can be used to develop valid behavioural detection and identification algorithms. This has implications for fostering wider collaborations between social sciences and health disciplines, which can both benefit from improved methods of measuring daily activities.

### Strengths and Limitations

The obvious limitation to these findings is the small, unrepresentative sample. This also prevented analysis by demographic characteristics. The sample was highly educated and located in a single small city in the UK. However, it was deemed adequate for the primary study aim of determining the feasibility of the wearable camera and reconstruction interview method. Further, we only investigated 11 relatively course activity types (for example cycling and driving were combined as transportation). It is possible that investigation at a more granular level would reveal important differences between self-report and camera reconstructions. This should be assessed in future studies with larger samples and more activity records.

The proposed method is still resource intensive. The cameras cost £300 each (as at November 2014) and require significant researcher time to conduct the interviews, annotate the images and reconstruct the day’s activities. However, the software used to download, view and annotate the images is open source and free to download [23]. In addition, we believe the reported method represents an improvement on direct researcher observation in terms of time and resources. Further, we believe that participants are more likely to behave ‘normally’ while wearing a camera compared with being directly observed by a researcher who is present with them. Indeed several participants reported that they often forget they were wearing the camera.

The fact that participants review their own time-stamped photos in the researcher-guided interview is one of the strengths of the method. Much device-based behavioural assessment has the researcher (or their analysis algorithms) viewing the data in isolation. In our method, ambiguity about the nature of daily activities is reduced and new information is revealed.

However, it is still a limitation that we rely on participant memory wherever the image data are missing (time at end of each day when camera is off, when lens is covered or light too low, or if removed during the day). We will incorporate the evidence from the wrist-worn accelerometer instruments in the next stage of our method development, as an additional objective indicator of behaviour collected when the camera is not operating [25].

Participants reported satisfaction with the protocol and considered it acceptable. One important issue is the reactions of third parties whose image is recorded. This may constrain the types of people willing to participate in these studies, and in turn limit the generalizability of any findings. This will be investigated in future work.

### Future Study

We make the following recommendations for future research:

- The study should be repeated on a larger and more representative sample to assess the validity of time-use diary data. The within-group reliability and test-retest reliability of these data will be important. The inter-rater reliability of researcher interviews, image annotation and day ‘reconstruction’ should also be assessed. Issues around recruitment and representativeness of sample should also be studied.
- This method should be used to assess the types of activities that are under-reported or missed by 24-hour time use diaries (because the participant does not view them as important, or they are too short to fit in the 10 minute event structure of HETUS-type instruments). Such activities might include tea or toilet breaks, simultaneous activities reported disproportionately as ‘secondary’, or using mobile devices that may not be adequately reported in diary formats.
- The use of objective behavioural data for the calibration of device algorithms (e.g. for accelerometers) is a promising application. Inter-disciplinary collaboration of sociology and public health in the investigation of health behaviours and of outcomes such as energy expenditure is an important by-product of this work.

## Conclusion

Valid and reliable measurement is fundamental to the sociological study of time use and activity budgets. This study demonstrates the feasibility of a new reference method that can be employed in validation, calibration and method comparison studies.
